# Exploring the Trajectory of Swallowing Within Psychomotor Development in Spinal Muscular Atrophy: Moving Toward Integrated Care

**DOI:** 10.3390/audiolres15050131

**Published:** 2025-10-07

**Authors:** Sofia Gandolfi, Claudia Dosi, Stefano Parravicini, Maria Teresa Arnoldi, Riccardo Zanin, Sofia Biagi, Livia Rinaldi, Riccardo Masson

**Affiliations:** 1 Developmental Neurology Unit, Fondazione IRCCS Istituto Neurologico Carlo Besta, 20133 Milan, Italy; sofia.gandolfi@istituto-besta.it (S.G.); claudia.dosi@istituto-besta.it (C.D.); mariateresa.arnoldi@istituto-besta.it (M.T.A.); riccardo.zanin@istituto-besta.it (R.Z.); sofia.biagi@istituto-besta.it (S.B.); riccardo.masson@istituto-besta.it (R.M.); 2 Department of Innovation Medicine and Engineering, University of Verona, 37134 Verona, Italy

**Keywords:** swallowing, spinal muscular atrophy, dysphagia, follow-up, bulbar function

## Abstract

Background: Spinal Muscular Atrophy type 1 (SMA type 1) is a genetic neuromuscular disease that typically presents before 6 months of age and is characterized by profound hypotonia, progressive muscle weakness, and early involvement of respiratory and bulbar musculature. Swallowing impairment (dysphagia) is a hallmark of SMA type 1 and significantly contributes to morbidity. Despite the documented benefits of disease-modifying therapies (DMTs) in terms of enhanced survival and motor outcomes, their impact on swallowing remains understudied. Aim: This study aims to longitudinally characterize swallowing function in children with SMA type 1 treated with DMTs, while contextualizing these findings in relation to the patients’ current motor abilities and cognitive performance. Materials and Methods: A single-center, longitudinal, observational study was conducted at IRCCS Besta, Milan, Italy, from 2021 to 2025. Swallowing function was evaluated using four validated scales (MAS, OrSAT, FILS, and p-FOIS), while motor and cognitive functions were assessed using CHOP-INTEND and age-appropriate cognitive tests (DQ/IQ). Patients were stratified by baseline swallowing status, pharmacological therapy, and age at DMT administration. Non-parametric statistical tests were applied. Results: No statistically significant changes in swallowing function were observed over one year in the overall cohort or its subgroups, despite significant improvements in motor function. MAS/e, FILS, and p-FOIS showed moderate associations with CHOP-INTEND and DQ/IQ scores. Conclusions: Swallowing function in children with SMA type 1 remained largely stable, while motor function significantly improved over one year, regardless of baseline swallowing status, DMT type, and age at administration. These findings underscore the need for standardized, longitudinal assessments of swallowing, motor, and cognitive functions in the management of SMA type 1.

## 1. Introduction

Spinal muscular atrophy (SMA) is a genetic neuromuscular disease characterized by the progressive degeneration of spinal and bulbar motor neurons. The primary genetic etiology of SMA is a homozygous deletion of the *SMN1* gene, leading to deficient production of the survival motor neuron (SMN) protein, which is essential for motor neuron survival and function [1,2].

Werdnig-Hoffmann disease, also known as spinal muscular atrophy type 1 (SMA type 1), is the most prevalent and severe form of SMA, with symptoms onset occurring before 6 months of age [3]. It is characterized by profound hypotonia, progressive muscle weakness, and early involvement of respiratory and bulbar muscles [4]. Prior to the availability of disease-modifying treatments (DMTs), SMA type 1 was associated with significant morbidity and early mortality, typically within the first two years of life.

Bulbar dysfunction, particularly swallowing impairment (dysphagia), is a defining feature of SMA type 1, typically manifesting within the first 12 months of life [1,5,6,7,8]. Dysphagia carries a significant risk of malnutrition, aspiration pneumonia, and failure to thrive [9,10,11]. Therefore, early and comprehensive assessment of swallowing function is essential for the timely implementation of nutritional and respiratory support interventions.

The advent of DMTs—including nusinersen (an antisense oligonucleotide), onasemnogene abeparvovec (a gene replacement therapy), and risdiplam (a small molecule splicing modifier of *SMN2*)—has fundamentally transformed the clinical trajectory of SMA type 1 by enabling prolonged survival and improved motor function outcomes [12]. However, the therapeutic impact on bulbar function, particularly swallowing ability, remains incompletely characterized. While preliminary reports suggest stabilization or modest improvements in oral feeding abilities [13,14], other studies document persistent or progressive dysphagia [9,15], underscoring the critical need for systematic longitudinal evaluation.

Despite the well-established importance of dysphagia management in SMA type 1, standardized protocols for serial swallowing assessments remain lacking. Current practice shows considerable variability in assessment frequency, evaluation methods, and clinical triggers for reassessment, which may lead to delayed therapeutic interventions and suboptimal patient outcomes.

This study aims to characterize the longitudinal evolution of swallowing function in children with SMA type 1, while contextualizing these findings within the patients’ current motor abilities and cognitive performance. Our first hypothesis is that DMTs have a significant impact on swallowing function, as measured by standardized and specific evaluation scales. Furthermore, we hypothesize that the interplay between functional domains may be relevant in the recovery of feeding abilities.

## 2. Materials and Methods

### 2.1. Study Design and Setting

This is a single-center, longitudinal, observational study aiming to examine swallowing function in pharmacologically treated SMA type 1 patients. Assessments were conducted between September 2021 and June 2025 at the SFERA (Swallowing and Feeding Rehabilitation and Assessment) outpatient clinic of the Fondazione IRCCS Istituto Neurologico Carlo Besta, Milan, Italy.

All assessments were conducted as part of routine clinical practice for patients with SMA type 1 at our center. Motor and cognitive evaluations were included if they were performed during the same follow-up visit as the swallowing assessment, or within a short temporal proximity to it.

### 2.2. Data Collection and Ethical Considerations

Demographic characteristics, clinical data, DMTs information, and motor milestone development were systematically collected during routine assessments and recorded in an anonymized database. All procedures adhered to Good Clinical Practice guidelines, the 1964 Helsinki Declaration, and its subsequent amendments [16]. The study was approved by the Institutional Ethics Committee of the Fondazione IRCCS Istituto Neurologico Carlo Besta (CET 27/25).

### 2.3. Patient Inclusion and Stratification

All SMA type 1 patients who were regularly followed up at our clinic were screened for participation in the study. To be classified as SMA type 1, patients had to present with motor symptom onset before 6 months of age and have a confirmed molecular diagnosis of SMA [3].

Children were eligible if they had undergone at least two swallowing assessments, with a minimum interval of 12 months between them. We established a threshold of 9 months for the first swallowing assessment to enable oral feeding across all consistencies [17]; only evaluations performed after the initiation of MDTs were included.

All other SMA types, as well as patients with only a single swallowing assessment, were excluded from the study.

Based on age at symptom onset, children were classified into three groups [18]: SMA type 1a (before one month), SMA type 1b (between one and three months), and SMA type 1c (between three and six months). We further distinguished patients into “early-treated” and “late-treated” groups, defined by the conventional threshold of 6 months between symptom onset and initiation of DMTs. Finally, children were classified based on their pre-treatment swallowing status (normal versus impaired) and sitting ability (sitters versus non-sitters).

### 2.4. Swallowing Assessment Protocol

Swallowing assessments were conducted using four validated swallowing scales: Mealtime Assessment Scale (MAS) [19,20], Oral and Swallowing Abilities Tool (OrSAT) [21], Food Intake LEVEL Scale (FILS) [22], and Paediatric Functional Oral Intake Scale (p-FOIS) [15].

MAS assesses the safety and efficacy of swallowing during a meal and includes four subscales addressing structural, functional, and environmental factors influencing the meal, as well as swallowing safety and efficacy. The safety score ranges from 0 to 12, and the efficacy score from 0 to 18, with higher scores (or percentages) indicating less safe or less effective swallowing [20].

OrSAT is a simple, validated tool for assessing oral motor function and swallowing in infants with SMA type 1. It consists of a checklist of abilities, with the maximum score varying according to the patient’s age. Functional impairment is categorized into four levels, ranging from no impairment to severe disorder. The tool was originally validated in children aged 0–2 years, but its use has been extended to include children up to 4 years [9]. To facilitate age-independent comparisons, OrSAT scores were expressed as a percentage of the maximum age-appropriate score, an approach not previously reported in the literature.

FILS is a 10-point observer-rated scale that measures the severity of dysphagia [22]. Scores of 1–3 indicate no oral intake, while scores above 4 reflect partial or total oral intake.

p-FOIS is a 6-point scale assessing changes in functional eating abilities [15]. Scoring is based on clinical observation and the patient’s habitual eating patterns. Scores 2–6 correspond to increasing levels of partial or total oral intake, whereas score 1 indicates no oral intake.

In line with standard clinical practice, all mealtime sessions were videotaped to ensure accurate scoring.

### 2.5. Motor and Cognitive Assessment

Motor function was evaluated using the Children’s Hospital of Philadelphia Infant Test of Neuromuscular Disorders (CHOP-INTEND) scale [23] during the routine multidisciplinary evaluations. The CHOP-INTEND was developed and validated for children up to 2 years of age; however, consistent with common practice in this population, it was also administered to older patients. Children were classified as “sitters” based on Item 26 of the Bayley-III gross motor scale [24], which assesses the ability to maintain an independent sitting position for 30 seconds.

Cognitive and developmental assessments are a routine part of clinical practice for SMA type 1 patients at our Center. They were evaluated using the Bayley Scales of Infant and Toddler Development, Third Edition (Bayley-III) [24], the Wechsler Preschool and Primary Scale of Intelligence, Fourth Edition (WPPSI-IV) [25], or the Wechsler Intelligence Scale for Children, Fourth Edition (WISC-IV) [26], according to the patients’ age. We included a single cognitive assessment for each patient, performed as close as possible to the first swallowing assessment (T0).

For both motor and cognitive function assessments, only the total score of the respective scale was considered.

### 2.6. Statistical Analysis

Being an exploratory pilot study, we included in the analysis all patients followed at our institute who met the eligibility criteria. No power analysis was performed to define the sample size.

Statistical analyses were performed using SPSS Statistics (version 28.0, IBM SPSS Inc., Chicago, IL, USA). Data normality was assessed using the Shapiro–Wilk test. Scale data that were not normally distributed are expressed as the median (range/interquartile range [IQR]). Nominal and categorical variables are presented as frequency (percentage [%]). Due to either the non-normal distribution or the ordinal nature of the variables, data were analyzed using non-parametric tests. The Wilcoxon signed-rank test was used to evaluate changes in swallowing function scores from baseline (T0) to the one-year follow-up (T1) for the same subjects, both for the entire sample and for different subgroups. To evaluate differences in the magnitude of change (T1-T0) between subgroups, the Mann–Whitney U test and Kruskal–Wallis test were applied. Due to the presence of a high number of tied ranks and an unbalanced sample size between subgroups, the exact two-tailed significance value was reported, as it provides a more accurate estimate of statistical significance under these conditions. Spearman’s rank correlation was used for all associations; the magnitude of correlation coefficients was interpreted according to Schober, Boer, and Schwarte’s classification [27]. For all statistical analyses significance was set at *p* < 0.05.

## 3. Results

This longitudinal study evaluated swallowing function in 41 children with SMA type 1 who met the inclusion criteria: a confirmed diagnosis of SMA type 1 and at least two swallowing assessments spaced by at least 12 months.

**Figure 1:**
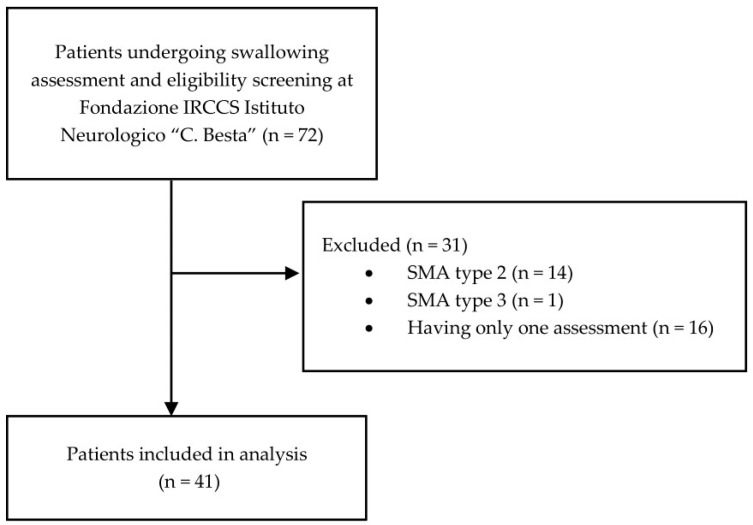
Patients eligibility flowchart.

Fifteen patients were excluded because they had a different SMA type, and 16 SMA type 1 patients were excluded because of the absence of a follow-up assessment (Figure 1).

Among the included patients, 38 (92.7%) carried two *SMN2* copies, while 3 children (7.3%) had three *SMN2* copies.

Patients received nusinersen (16/41, 39%), risdiplam (8/41, 19.5%), or onasemnogene abeparvovec (17/41, 41.5%) as their initial treatment.

Comprehensive demographic and clinical characteristics of the study population are presented in Table 1.

The results of longitudinal swallowing assessments (MAS, OrSAT, FILS, FOIS) and CHOP-INTEND scores are presented in Table 2. Follow-up OrSAT data were available for only 18 patients, as the remaining children were older than four years at T0 or T1. A second CHOP-INTEND assessment (T1) was not available for one patient; therefore, only 40 children were included in this analysis.

A developmental/cognitive assessment was performed in 31/41 children (75.6%) at T0, at a median age of 32.9 months (range: 18.0–118.8). The median score—Developmental Quotient (DQ) or Intelligence Quotient (IQ)—was 85 (IQR: 29; range: 55–120).

No statistically significant differences were observed within the entire group between baseline (T0) and one-year follow-up (T1) assessments for any of the swallowing scales. In contrast, motor function, as measured by the CHOP-INTEND, showed a significant improvement (*p* < 0.001) (see Table A1 in Appendix A). Similarly, no significant differences in any of the swallowing scales were found between T0 and T1 when patients were stratified by treatment timing or type (Table A2 and Table A3 in Appendix A). OrSAT scores could not be calculated for late-treated patients due to age-related limitations. On the other hand, a significant improvement in motor function was observed in the early-treated group.

No statistically significant changes were observed in any of the swallowing-specific scales—OrSAT, MAS/e, MAS/s, FILS, or p-FOIS—after one year of follow-up, regardless of pre-treatment swallowing status, whether patients had adequate or impaired baseline function (Table A4, Appendix A). Conversely, CHOP-INTEND scores increased significantly over the same period in both groups.

Finally, no statistically significant changes in swallowing abilities were detected either in patients unable to maintain a sitting position or in sitters, whereas CHOP-INTEND scores significantly improved between T0 and T1 among sitters (Table A5 in Appendix A).

CHOP-INTEND scores were significantly correlated with swallow efficacy (MAS/e) and swallowing observer-rating scales (p-FOIS and FILS) at both T0 and T1, whereas no association was found with OrSAT and MAS/s (Table 3).

Furthermore, a moderate association was observed between developmental/cognitive function (DQ/IQ) and motor function, as well as between cognitive function and swallow efficacy (MAS/e), and between cognitive function and p-FOIS (Table 4).

No association was found between patients’ age and changes in swallowing scale scores from T0 to T1 (Table A6 in the Appendix A).

## 4. Discussion

To our knowledge, this is the first longitudinal observational study to quantitatively assess the development of swallowing function in children with SMA type 1, using a standardized mealtime assessment performed by speech therapists (MAS), in addition to other clinical tools validated in SMA (OrSAT, p-FOIS). The use of clinical assessments is valuable, providing a simple, non-invasive, universally applicable, and easily replicable method for follow-up. This approach reduces reliance on instrumental assessments, which can be reserved for patients exhibiting overt signs of dysphagia during mealtime evaluation or presenting with complex clinical profiles that may limit the sensitivity of clinical assessment.

The study findings indicate overall stability in swallowing function after at least 12 months of follow-up, with no significant improvement or deterioration detected, regardless of pre-treatment swallowing status, sitting ability, treatment timing, or DMT administered. These results are consistent with previous qualitative studies [9,10,13,15,28,29,30,31,32,33,34,35,36,37,38,39,40,41,42,43] which demonstrated the positive impact of DMTs on dysphagia, and reinforce the evidence for the efficacy of DMTs in mitigating or preventing the deterioration of swallowing, which typically occurs early in the natural history of SMA type 1. However, heterogeneity in outcome measures limits direct comparisons across studies, with some reporting only the initiation of enteral nutrition as a marker of swallowing decline [10,30,31,33,34,35], while others include clinical and/or instrumental assessments [9,13,15,28,29,37,38,39,40,41,42,43].

In contrast to the natural history of SMA type 1—wherein children typically lose oral feeding ability within the first year of life, with the nature and frequency of deficits varying according to phenotype severity [9,11,21,44,45,46,47,48]—treated children appear to maintain their baseline swallowing function throughout the follow-up period, regardless of the age at assessment.

We consider these findings fairly representative of the broader SMA type 1 population, as, in line with previous research, we observed significant improvements in motor function [5,12,28,37,49], particularly in “early-treated” children. This observation supports the growing consensus that early intervention is crucial for maximizing motor outcomes in SMA type 1 [35].

Moreover, these data suggest that while DMTs can positively impact motor function, their effect on swallowing appears primarily to stabilize the pre-treatment status—preventing deterioration—rather than induce improvement. This dissociation may reflect distinct pathophysiological mechanisms affecting motor versus bulbar muscles, differences in the tissue distribution and efficacy of pharmacological treatments, or variability in the sensitivity of assessment tools and rehabilitation protocols. In fact, all children in this cohort received regular physiotherapy, whereas only two of them underwent targeted swallowing interventions (Table 2). This imbalance in rehabilitative focus may contribute to the observed divergence in functional outcomes. While the role of motor rehabilitation is well recognized and routinely integrated into standard care, the impact of swallowing rehabilitation remains underexplored in this population. Further studies are warranted to evaluate whether systematic, individualized dysphagia interventions could enhance swallowing function, particularly in the context of early pharmacological treatment.

A significant association was found between swallowing efficacy (MAS/e, pFOIS) and motor function, but this was not observed for swallowing safety (MAS/s) and OrSAT, likely reflecting the involvement of different pathophysiological mechanisms affecting distinct aspects of swallowing.

Moderate associations between cognitive function and both motor and swallowing efficacy scores suggest that developmental/cognitive status may influence or reflect overall neuromuscular function and disease severity in SMA type 1. The impact of SMA type 1 on cognitive function remains debated. While we are fully aware of the complexity involved in assessing cognition in children with motor disabilities, cognitive function may play a role in the development of compensatory strategies, not only in the motor domain but also in swallowing. The interrelationships among these domains warrant further investigation, as cognitive development may be influenced by disease burden, nutritional status, or the effects pharmacological therapy.

Finally, additional research is needed to examine in depth the evolution of cognitive, motor, and swallowing functions in children identified through newborn screening. Early identification enables the initiation of treatment during a critical developmental window, potentially altering the natural history of the disease. However, the long-term impact of early treatment on the interplay between cognitive, motor, and bulbar functions remains poorly understood. A better understanding of these trajectories could inform future clinical practice and care planning, guiding the design of interdisciplinary interventions aimed at optimizing developmental outcomes.

### Limitations

A limitation of this study is the relatively small sample size in subgroup analyses and the heterogeneity in patients’ ages at the time of the swallowing assessments. Swallowing abilities develop physiologically during early childhood, with toddlers typically able to manage adult-like solid foods between two and three years of life [17]. In our sample, 29% of children were under 2 years of age at the first assessment; therefore, their ability to handle all food consistencies may have evolved over the course of the follow-up. Future studies should more precisely investigate the development of swallowing abilities stratifying patients according to age, in order to achieve greater stability and generalizability of the findings. Moreover, the median follow-up duration was 12 months, which may be too short to fully capture meaningful changes in motor and swallowing functions. Longer follow-up studies are warranted to better characterize the progression of cognitive, swallowing, and motor abilities in this population.

In addition, this study relied exclusively on clinical scales to evaluate swallowing function but did not include objective instrumental assessments. While clinical scales are non-invasive, provide valuable information in daily practice and enable standardized comparisons across patients, they cannot fully capture the physiological mechanisms underlying swallowing impairment. Future studies integrating both clinical and instrumental tools could therefore offer a more comprehensive understanding of swallowing function and its evolution in this population.

## 5. Conclusions

This study demonstrates that while motor function significantly improves with early treatment, swallowing function remains stable for at least a one-year period, regardless of the DMT administered. These findings highlight the need for comprehensive management strategies that target both motor and bulbar functions to optimize patient outcomes. Early intervention remains essential for motor improvement and the preservation of swallowing abilities.

## Figures and Tables

**Table 1:** Clinical characteristics of the study population.

Demographics and Clinical Characteristics	n = 41
Gender, n (%)	
Male	15 (36.6)
Female	26 (63.4)
Enteral feeding, n (%)	
Yes	12 (29.3)
No	29 (70.7)
Age at symptoms onset, months, median (range)	1.7 (0.0–6.0)
SMA 1 subtype, n (%)	
SMA type 1a	17 (41.5)
SMA type 1b	16 (39.0)
SMA type 1c	8 (19.5)
Age at DMT initiation, months, median (range)	4.5 (1.0–42.1)
Symptoms onset to DMT timespan, months, median (range)	2.7 (0.5–37.1)
Pre-treatment swallowing status, n (%)	
Normal	27 (65.9)
Impaired	14 (34.1)
Age at first assessment (T0), months, median (range)	38.9 (9.8–114.3)
Age at second assessment (T1), months, median (range)	55.3 (24.9–126.3)
Time between T0 and T1, months, median (range)	13.0 (12.0–24.6)
Swallowing rehabilitation, n (%)	
Yes	2 (4.9)
No	39 (95.1)

**Table 2:** Descriptive statistics for MAS, OrSAT, FILS, p-FOIS, and CHOP-INTEND at first (T0) and second (T1) swallowing assessment.

Scale	n	Median (IQR; Range) at T0	Median (IQR; Range) at T1	Score Range
MAS	41			
MAS/s		1 (4; 0–12)	2 (6; 0–12)	0–12
MAS/e		2 (9; 0–18)	2 (11; 0–18)	0–18
OrSAT ^1^	18	95.8 (27.1; 8.3–100.0)	91.7 (18.8; 8.3–100.0)	0–100
FILS	41	8 (2; 2–10)	8 (6; 2–10)	1–10
p-FOIS	41	5 (2; 1–6)	5 (4; 1–6)	1–6
CHOP-INTEND	40	54 (9; 24–64)	56 (7; 22–64)	0–64

^1^ To compare patients of different ages, we reported the OrSAT score as a percentage of the maximum score for each patient’s age.

**Table 3:** Association between swallowing scales and CHOP-INTEND scores at T0 and T1.

Swallowing Scale	T0 CHOP-INTEND		T1 CHOP-INTEND	
	r_s_	*p*-Value	r_s_	*p*-Value
OrSAT	0.252	0.205	0.245	0.327
MAS				
MAS/s	−0.234	0.146	−0.271	0.091
MAS/e	**−0.414**	0.008 **	**−0.498**	0.001 **
FILS	**0.348**	0.028 *	**0.426**	0.005 **
p-FOIS	**0.364**	0.021 *	**0.438**	0.005 **

Spearman’s rank correlation was used to assess the strength and direction of the monotonic association between motor scores and swallowing scales scores at T0 and T1 separately. The magnitude of the correlation coefficients was interpreted according to the classification proposed by Schober, Boer, and Schwarte [27]. *: statistically significant with *p* < 0.05; **: statistically significant result with *p* < 0.01

**Table 4:** Association between swallowing/motor function and cognitive development at T0.

Assessment Scale	Developmental (DQ)/ Cognitive Function (IQ)	
	r_s_	*p*-Value
OrSAT	0.446	0.064
MAS		
MAS/s	−0.244	0.185
MAS/e	**−0.443**	0.012 *
FILS	0.317	0.082
p-FOIS	**0.382**	0.034 *
CHOP-INTEND	**0.590**	<0.001 **

Spearman’s rank correlation was used to assess the strength and direction of the monotonic association between motor, swallowing, and cognitive scales scores at T0. The magnitude of the correlation coefficients was interpreted according to the classification proposed by Schober, Boer, and Schwarte [27]. *: statistically significant with *p* < 0.05; **: statistically significant result with *p* < 0.01.

## Data Availability

The original contributions presented in the study are included in the article, further inquiries can be directed to the corresponding author.

## Abbreviations

The following abbreviations are used in this manuscript:

CHOP-INTEND	Children’s Hospital of Philadelphia Infant Test of Neuromuscular Disorders
DQ	Developmental Quotient
FILS	Food Intake LEVEL Scale
IQ	Intelligence Quotient
IQR	Interquartile range
MAS	Mealtime Assessment Scale
OrSAT	Oral and Swallowing Abilities Tool
p-FOIS	Paediatric Functional Oral Intake Scale
SFERA	Swallowing and Feeding Rehabilitation and Assessment
SMA	Spinal Muscular Atrophy
SMA type 1	Spinal Muscular Atrophy type 1
SMN	Survival Motor Neuron
T0	Baseline
T1	One-year follow-up
WISC-IV	Wechsler Intelligence Scale for Children, Fourth Edition
WPPSI-IV	Wechsler Preschool and Primary Scale of Intelligence, Fourth Edition

## Appendix A

**Table A1:** Comparison of swallowing function scores between baseline (T0) and one-year follow-up (T1) in the whole population.

Test	n	Negative Ranks	Positive Ranks	Ties	Z	*p*-Value
OrSAT	18	5	6	7	−0.224	0.823
MAS						
MAS/s	41	11	13	17	−1.011	0.312
MAS/e	41	10	20	11	−1.059	0.290
FILS	41	6	8	27	−0.289	0.772
p-FOIS	41	4	7	30	−0.189	0.850
CHOP-INTEND	40	**5**	**22**	**13**	**−3.503**	**<0.001 ****

The Wilcoxon signed-rank test was used to evaluate changes in swallowing function scores from baseline (T0) to the one-year follow-up (T1) within the same subjects. **: statistically significant result with *p* < 0.01.

**Table A2:** Comparison of swallowing function scores between baseline (T0) and one-year follow-up (T1) according to treatment timing.

Test	Treatment Group	n	Negative Ranks	Positive Ranks	Ties	Z	*p*-Value
OrSAT	Early	18	5	6	7	−0.224	0.823
MAS							
MAS/s	Early	37	9	12	16	−1.169	0.242
	Late	4	2	1	1	−0.577	0.564
MAS/e	Early	37	8	19	10	−1.152	0.249
	Late	4	2	1	1	0.000	1.000
FILS	Early	37	6	7	24	−0.464	0.643
	Late	4	0	1	3	−1.000	0.317
p-FOIS	Early	37	4	5	28	−0.312	0.755
	Late	4	0	2	2	−1.414	0.157
CHOP-INTEND	Early	37	**4**	**20**	**13**	**−3.636**	**<0.001 ****
	Late	3	1	2	0	0.000	1.000

The Wilcoxon signed-rank test was used to evaluate changes in swallowing function scores from baseline (T0) to the one-year follow-up (T1) within the same subjects, stratified according to treatment timing. **: statistically significant result with *p* < 0.01.

**Table A3:** Comparison of swallowing and motor function scores between baseline (T0) and one-year follow-up (T1) according to DMT.

Test	Treatment	n	Negative Ranks	Positive Ranks	Ties	Z	*p*-Value
OrSAT	N	7	3	2	2	−0.813	0.416
	O	11	2	4	5	−1.160	0.246
MAS							
MAS/s	R	8	5	1	2	−1.000	0.317
	N	16	4	6	6	−0.417	0.638
	O	17	2	6	9	−1.496	0.135
MAS/e	R	8	2	2	4	−0.730	0.465
	N	16	4	9	3	−0.950	0.342
	O	17	4	9	4	−1.077	0.282
FILS	R	8	0	2	6	−1.342	0.180
	N	16	3	3	10	−0.333	0.739
	O	17	3	3	11	−0.744	0.457
p-FOIS	R	8	0	1	7	−1.000	0.317
	N	16	1	4	11	−0.707	0.480
	O	17	3	2	12	−0.707	0.480
CHOP-INTEND	R	8	1	4	3	−1.625	0.104
	N	15	3	8	4	−1.568	0.117
	O	17	**1**	**10**	**6**	**−2.803**	**0.005 ****

The Wilcoxon signed-rank test was used to evaluate changes in swallowing function scores from baseline (T0) to the one-year follow-up (T1) within the same subjects, stratified according to DMTs. **: statistically significant result with *p* < 0.01.

**Table A4:** Comparison of swallowing function scores between baseline (T0) and one-year follow-up (T1) according to pre-therapy swallowing status.

Test	Pre-Therapy Swallowing Status	n	Negative Ranks	Positive Ranks	Ties	Z	*p*-Value
OrSAT	Normal	13	4	3	6	−0.171	0.865
	Impaired	5	1	3	1	−0.365	0.715
MAS							
MAS/s	Normal	27	7	5	15	−0.120	0.905
	Impaired	14	4	8	2	−1.339	0.181
MAS/e	Normal	27	5	13	9	−0.952	0.341
	Impaired	14	5	7	2	−0.629	0.529
FILS	Normal	27	4	7	16	−0.552	0.581
	Impaired	14	2	1	11	−1.069	0.285
p-FOIS	Normal	27	2	5	20	−1.134	0.257
	Impaired	14	2	2	10	−0.736	0.461
CHOP-INTEND	Normal	27	**2**	**14**	**11**	**−2.775**	**0.006 ****
	Impaired	13	**3**	**8**	**2**	**−2.050**	**0.040 ***

The Wilcoxon signed-rank test was used to evaluate changes in swallowing function scores from baseline (T0) to the one-year follow-up (T1) within the same subjects, stratified according to pre-DMT swallowing status. *: statistically significant with *p* < 0.05; **: statistically significant result with *p* < 0.01.

**Table A5:** Comparison of swallowing function scores between baseline (T0) and one-year follow-up (T1) according to sitting position.

Test	Sitting Position	n	Negative Ranks	Positive Ranks	Ties	Z	*p*-Value
OrSAT	Yes	16	4	6	6	−0.719	0.472
	No	2	1	0	1	−1.000	0.317
MAS							
MAS/s	Yes	33	10	8	15	−0.178	0.859
	No	8	1	5	2	−1.725	0.084
MAS/e	Yes	33	8	16	9	−0.390	0.697
	No	8	2	4	2	−1.581	0.114
FILS	Yes	33	5	8	20	−0.180	0.857
	No	8	1	0	7	−1.000	0.317
p-FOIS	Yes	33	3	5	25	−0.302	0.763
	No	8	1	2	5	0.000	1.000
CHOP-INTEND	Yes	33	**3**	**18**	**12**	**−3.414**	**<0.001 ****
	No	7	2	4	1	−1.051	0.293

The Wilcoxon signed-rank test was used to evaluate changes in swallowing function scores from baseline (T0) to the one-year follow-up (T1) within the same subjects, stratified according to their ability to maintain sitting position. **: statistically significant result with *p* < 0.01.

**Table A6:** Association between age at assessment and changes in swallowing scale scores at T0 and T1.

Swallowing Scale	r_s_	*p*-Value
OrSAT	−0.329	0.183
MAS/s	−0.244	0.159
MAS/e	−0.147	0.359
FILS	−0.035	0.829
p-FOIS	−0.039	0.808

Spearman’s rank correlation was used to assess the strength and direction of the monotonic association between the difference in swallowing scales scores at T0 and T1 and age at assessment. The magnitude of the correlation coefficients was interpreted according to the classification proposed by Schober, Boer, and Schwarte [27].
